# Supplementary material for: Illumina iSeq 100 and MiSeq exhibit similar performance in freshwater fish environmental DNA metabarcoding
Source: Sci Rep. 2021 Aug 3;11:15763. doi: 10.1038/s41598-021-95360-5 (PMC8333312; doi:10.1038/s41598-021-95360-5)
Supplement: Supplementary file 1 — Supplementary Information 1. [file 41598_2021_95360_MOESM1_ESM.docx]

**Supplementary Information for the article:**

**Illumina iSeq 100 and MiSeq exhibit similar performance in freshwater fish environmental DNA metabarcoding**

**Authors:**

**Ryohei Nakao^1*^, Ryutei Inui^2^, Yoshihisa Akamatsu^1^, Masuji Goto^3^, Hideyuki Doi^4^, Shunsuke Matsuoka^4^**

^1^Graduate School of Science and Technology for Innovation, Yamaguchi University, 2-16-1, Tokiwadai, Ube, Yamaguchi, 755-8611, Japan.

^2^Faculty of Socio-Environmental Studies, Fukuoka Institute of Technology, 3-30-1, Wajiro-higashi, Higashi-Ku, Fukuoka, 811-0295, Japan.

^3^Research and Development Center of Nippon Koei Co. Ltd., 2304, Inarihara, Tsukuba, Ibaraki, 300-1259, Japan.

^4^Graduate School of Simulation Studies, University of Hyogo, 7-1-28, Minatojima-minamimachi, Chuo-ku, Kobe, 650-0047, Japan.

**Corresponding Author**

*Ryohei Nakao. Graduate School of Science and Technology for Innovation, Yamaguchi University, 2-16-1, Tokiwadai, Ube, Yamaguchi, 755-8611, Japan.

lineck92@yahoo.co.jp

Supplementary Figure S1:

**Comparison of sequence quality between iSeq and MiSeq.**

Supplementary Figure S2:

**Relationship of sequence read per sample between iSeq and MiSeq after the merge pair step.**

Supplementary Figure S3:

**Relationship of sequence read per sample between iSeq and MiSeq after the quality filtering step.**

Supplementary Figure S4:

**Figure S4. Relationship of sequence read per sample between iSeq and MiSeq after the denoising step.**

Supplementary Figure S5:

**Figure S5. Relationship of remained sequence read per sample between iSeq and MiSeq for the taxonomic assignment.**

Supplementary Figure S6:

**Species accumulation curves of each samples in iSeq platform.**

Supplementary Figure S7:

**Species accumulation curves of each samples in MiSeq platform.**


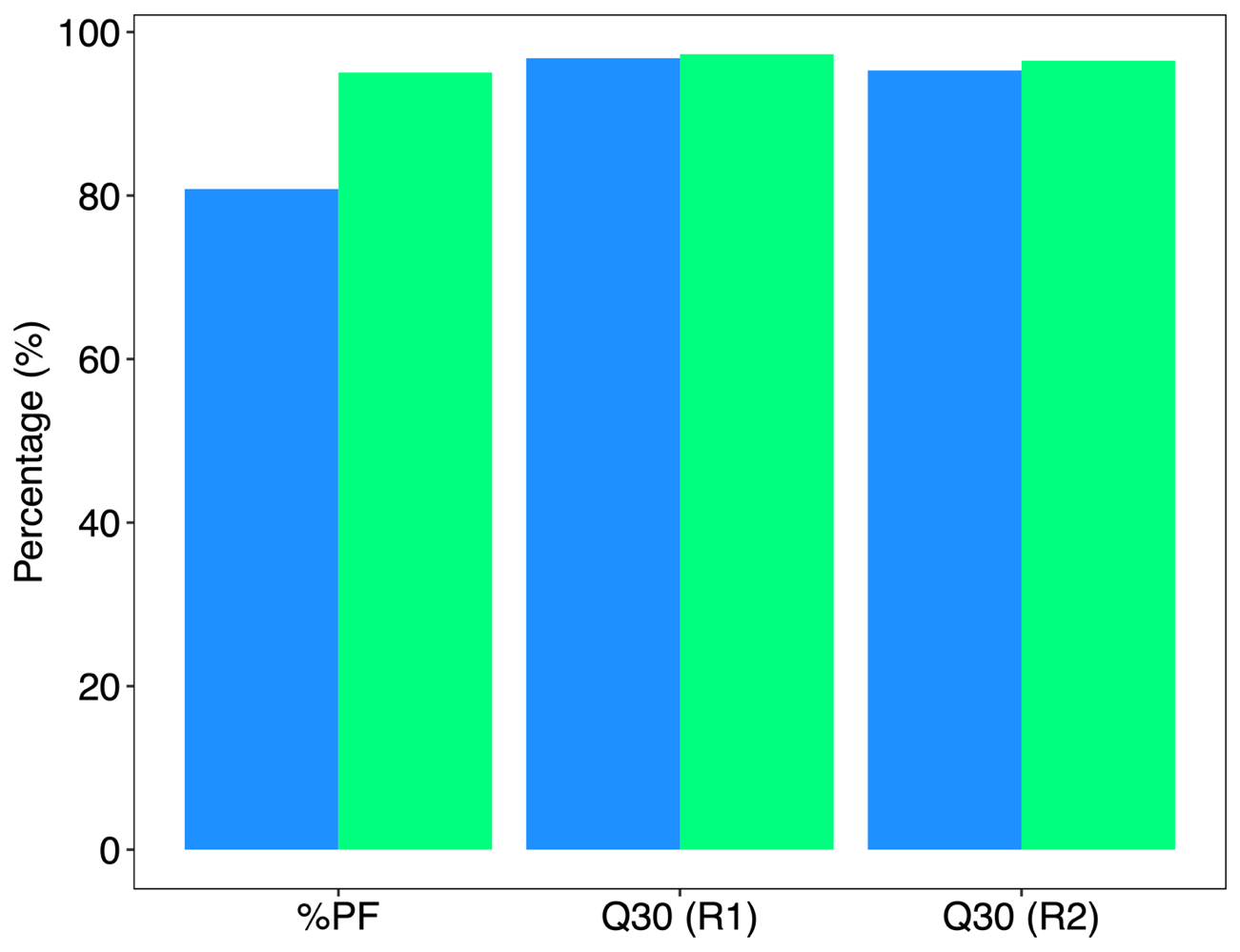


**Figure S1. Comparison of sequence quality between iSeq and MiSeq.** Blue and green bar plots show the results of iSeq and Miseq. Each bar also shows the percentage of pass filtering (80.8 vs. 95.1), Read 1 Q30 (96.8 vs. 97.3), and Read 2 Q30 (95.3 vs. 96.5), respectively. The bar plots were illustrated using “ggplot” function in ggplot2 package in R ver. 3.6.2.


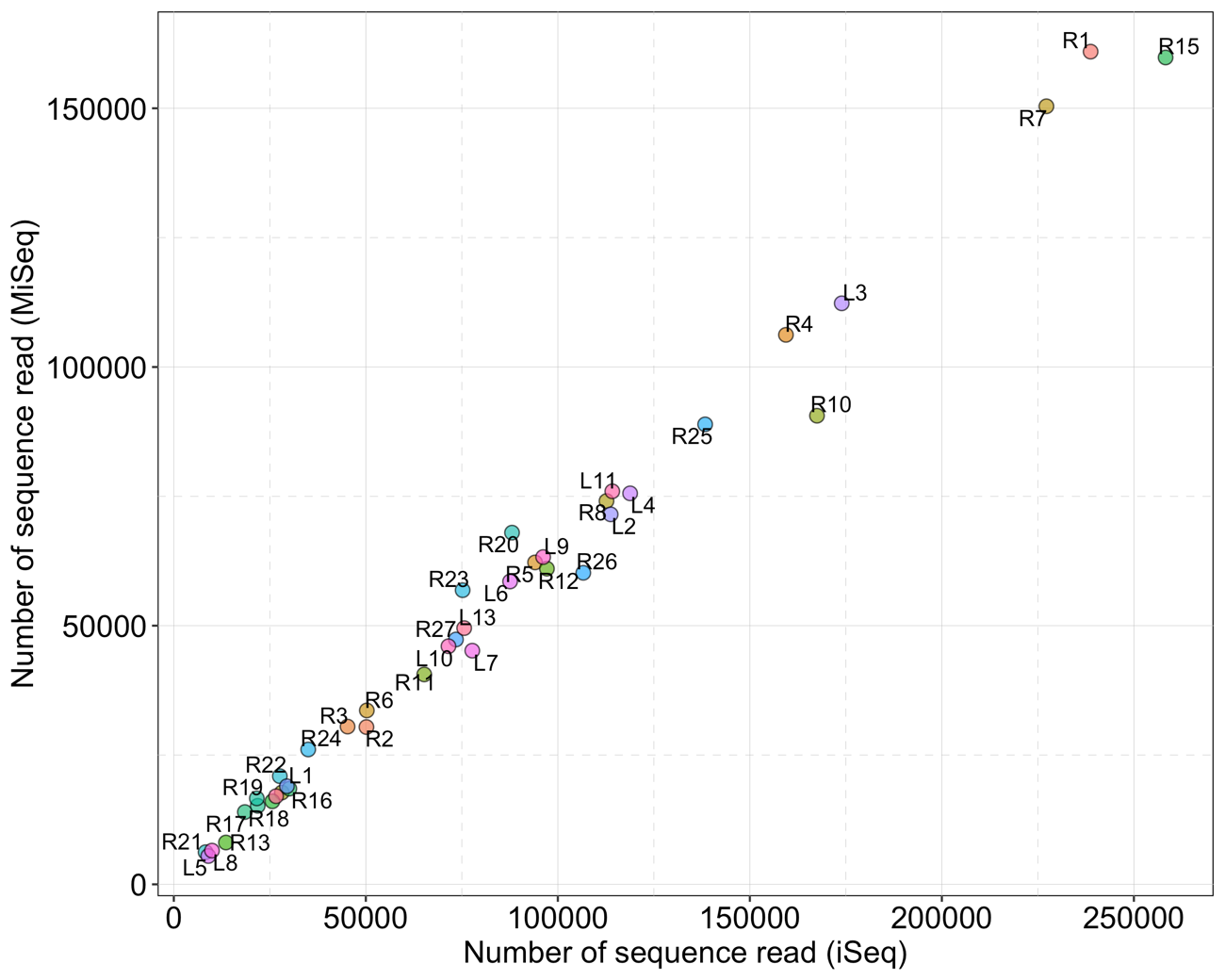


**Figure S2. Relationship of sequence read per sample between iSeq and MiSeq after the merge pair step.** The dot plot was illustrated using “ggplot” function in ggplot2 package in R ver. 3.6.2. There was significant positive correlation between the remained sequence reads of iSeq and MiSeq (spearman’s rank correlation, ρ = 0.991, *p* < 0.01)


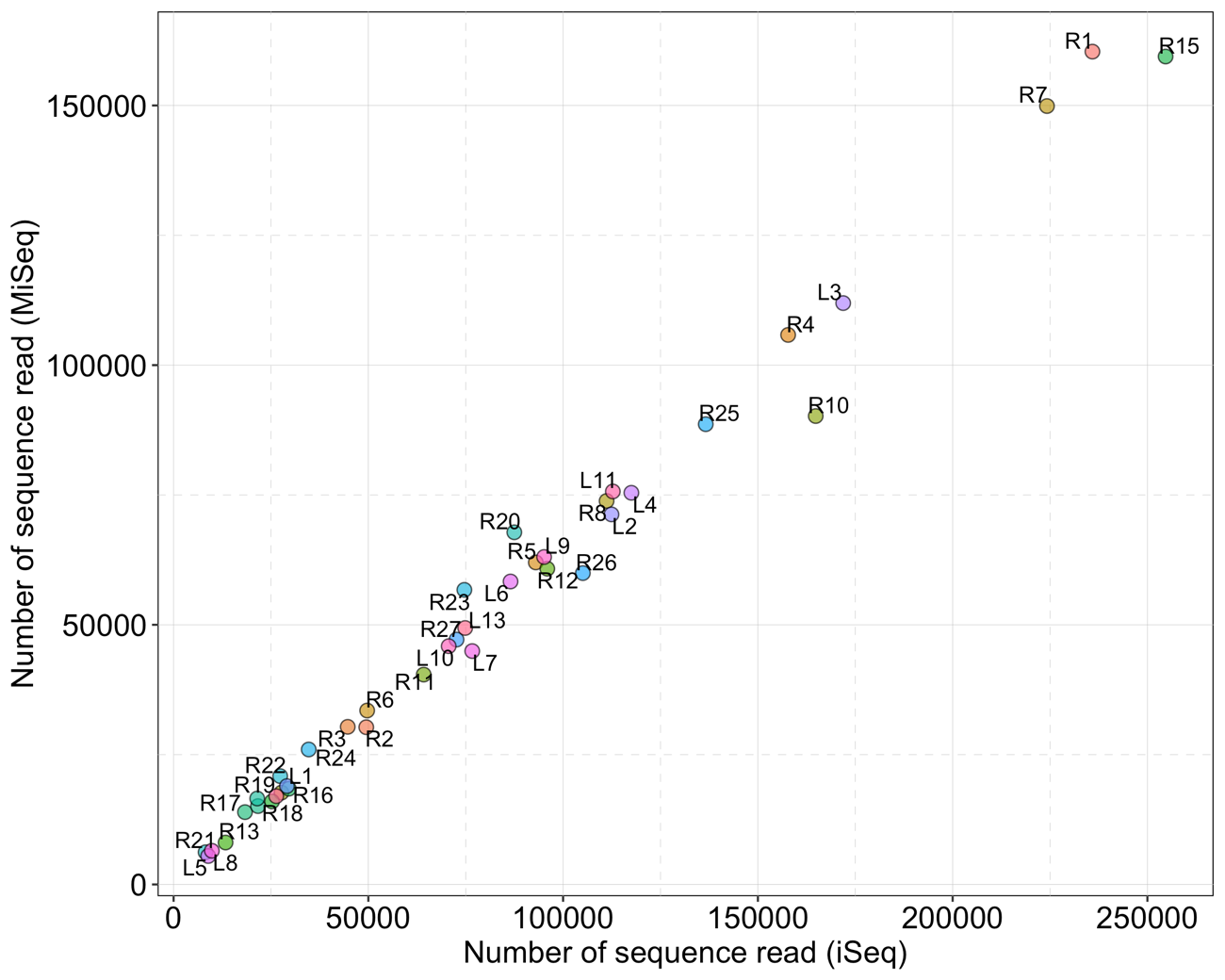


**Figure S3. Relationship of sequence read per sample between iSeq and MiSeq after the quality filtering step.** The dot plot was illustrated using “ggplot” function in ggplot2 package in R ver. 3.6.2. There was significant positive correlation between iSeq and MiSeq (spearman’s rank correlation, ρ = 0.991, *p* < 0.01)


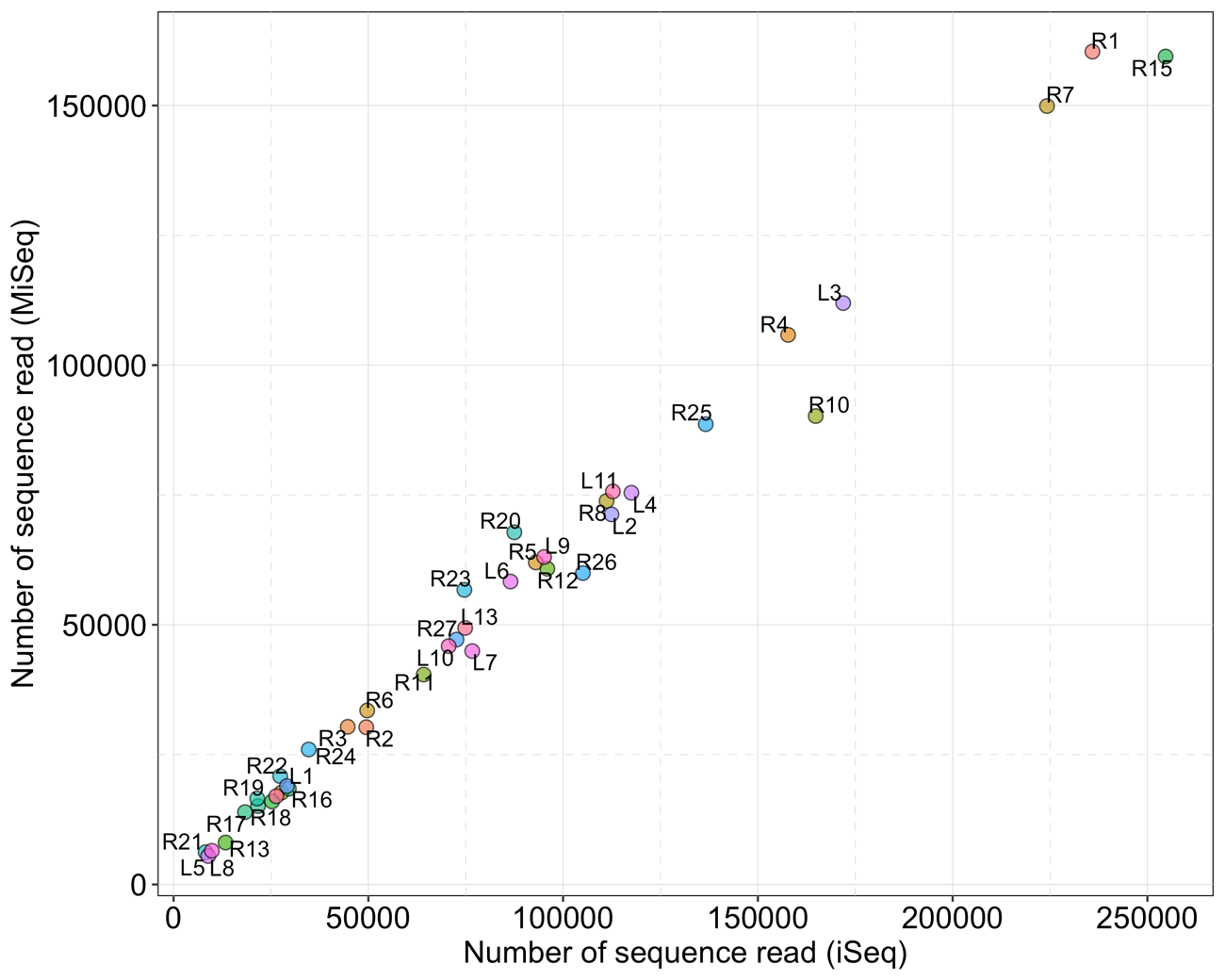


**Figure S4. Relationship of sequence read per sample between iSeq and MiSeq after the denoising step.** The dot plot was illustrated using “ggplot” function in ggplot2 package in R ver. 3.6.2. There was significant positive correlation between iSeq and MiSeq (spearman’s rank correlation, ρ = 0.993, *p* < 0.01)


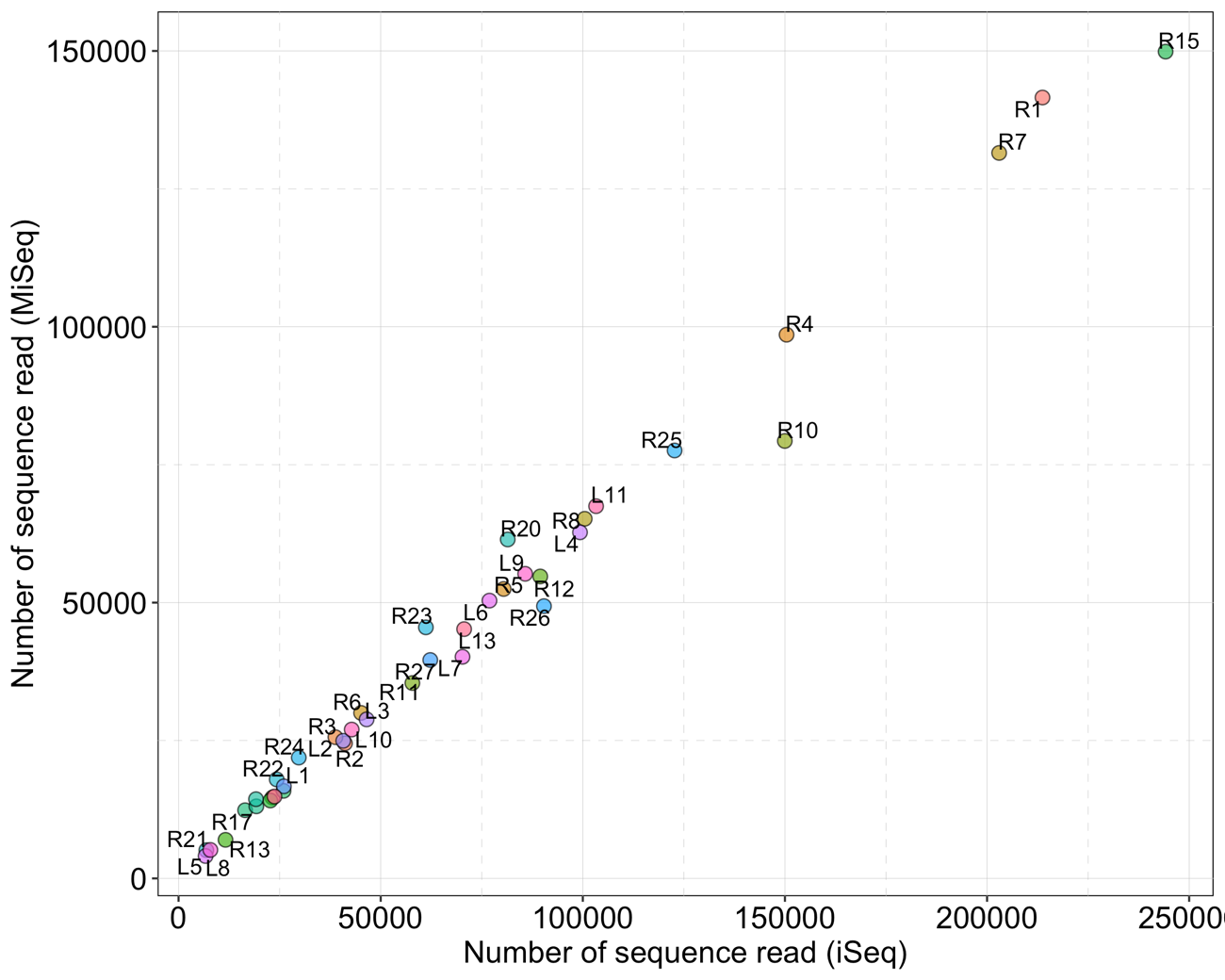


**Figure S5. Relationship of remained sequence read per sample between iSeq and MiSeq for the taxonomic assignment.** The dot plot was illustrated using “ggplot” function in ggplot2 package in R ver. 3.6.2. There was significant positive correlation between iSeq and MiSeq (spearman’s rank correlation, ρ = 0.993, *p* < 0.01).


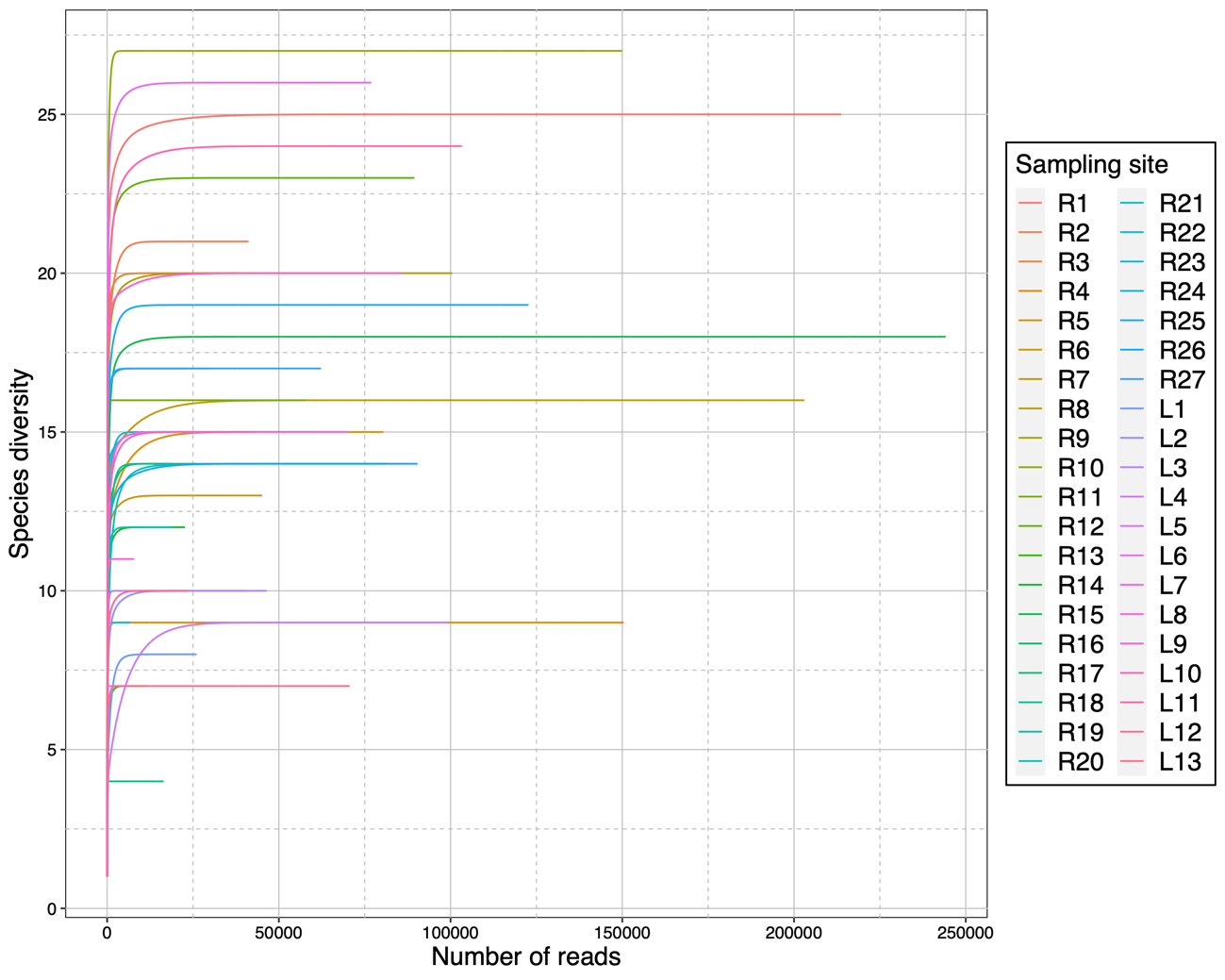


**Figure S6. Species accumulation curves of each samples in iSeq platform.** This graph was illustrated using “rarecurve” and “ggplot” function in vegan and ggplot2 package in R ver. 3.6.2, respectively.


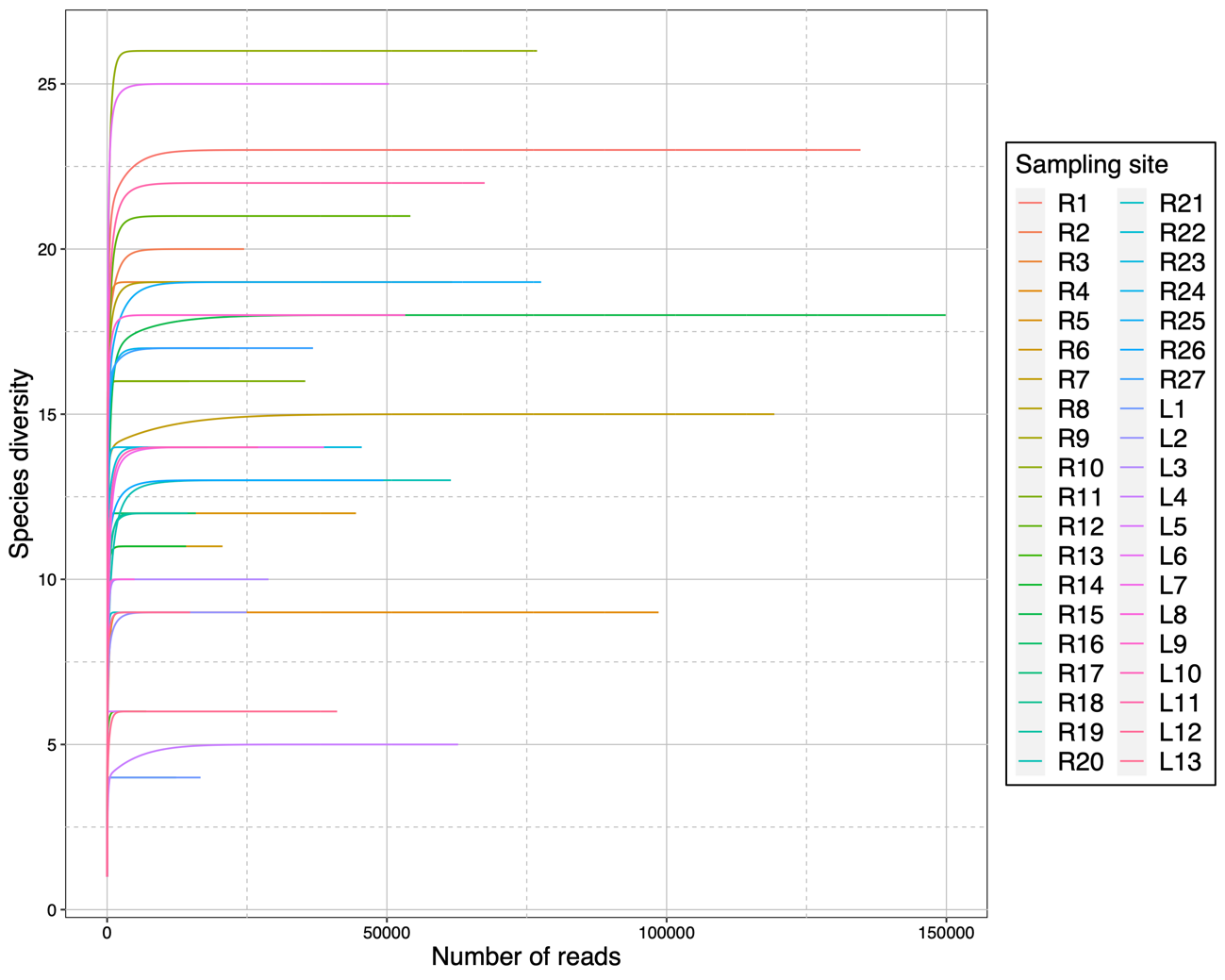


**Figure S7. Species accumulation curves of each samples in MiSeq platform.** This graph was illustrated using “rarecurve” and “ggplot” function in vegan and ggplot2 package in R ver. 3.6.2, respectively.
